# The relationship between spirituality and aggression in the workplace during the COVID-19 pandemic: A cross-sectional study among Iranian nurses

**DOI:** 10.1371/journal.pone.0279247

**Published:** 2022-12-21

**Authors:** Hossein Harati, Hossein Mohsenipouya, Nouraddin Mousavinasab, Alireza Sangani, Md. Khorshed Alam, Mohammed A. Mamun

**Affiliations:** 1 Faculty of Nursing, Mazandaran University of Medical Sciences, Sari, Iran; 2 Health Education and Promotion, Faculty of Nursing, Mazandaran University of Medical Sciences, Sari, Iran; 3 Department of Biostatistics, Faculty of Health, Mazandaran University of Medical Sciences, Sari, Iran; 4 Department of Cultural Psychopathology, Farabi, Psychological Sciences Research Center, Mazandaran, Iran; 5 Department of Information Management, The United Nations Refugee Agency (UNHCR), Dhaka, Bangladesh; 6 CHINTA Research Bangladesh, Dhaka, Bangladesh; 7 Department of Public Health and Informatics, Jahangirnagar University, Dhaka, Bangladesh; University of Tripoli, LIBYA

## Abstract

**Background:**

Aggression towards nurses in the workplace arises from various reasons, reportedly increasing during the COVID-19 pandemic. Where aggression can be maintained by spiritual well-being, as it is said that spirituality is a coping skill and psychological well-being maintainer–but there is little known, especially during the pandemic. Thus, this study explored the effect of spirituality on aggression among the nurses working in the COVID-19 wards.

**Methods:**

This cross-sectional data from 200 nurses involved in the COVID-19 patient treatment were collected using a random sampling method from four hospitals in East Mazandaran province, Iran. Responses were collected based on socio-demographics, Buss-Perry Aggression Questionnaire, and Paloutzian & Ellison Spiritual Well-being Scale. T-test, ANOVA, Pearson correlation coefficient, and multiple linear regression were applied for data analysis.

**Results:**

The mean age of nurses was 31.49±6.88 (range: 21–48) years. Nurses working in the COVID-19 wards have a mean score of spiritual health of 67.21±12.84 (out of 120), whereas 51.77±10.96 (out of 116) was for aggression. The results showed a significant negative weak correlation between aggression and spiritual health (r = -.285, *p*<0.01). As per regression analysis, spiritual health [β = -.264], age [β = -.374], and working experience [β = 4.156] were the significant factors associated with aggression (*p*<0.05).

**Conclusions:**

It is evident that nurses who consider spirituality in their life actions are in a state of reduced negative emotions, such as aggression. Thus, policymakers and managers of the healthcare settings are suggested to promote spirituality among the nurses through spiritual care education, providing the ground for promoting spirituality and a positive attitude towards it.

## 1 Introduction

The COVID-19 pandemic has changed the way of living worldwide, which seriously affects many people. By 27 November 2021, 261 million positive cases of COVID-19 were documented in Iran, including 5.19 million deaths. At the time of the pandemic, healthcare workers (HCWs) have tirelessly worked to combat the situation by involving direct management of the COVID-19 patients, which increases their risk of personal infection as well as psychological suffering from issues like fear, aggression, depression, stress, etc. [1–3]. Many HCWs are reported to die by suicide due to being infected with COVID-19, work‐related stress, and fear related to COVID‐19 infection or transmission [4]. Furthermore, aggression and violence account for the negative consequences on the health of patients and nurses health, and their safety and well-being; they are the major concerns in psychiatric inpatient care globally [5].

The word aggression originated in the early 17^th^ century, which is derived from the Latin word, ‘aggredi’, meaning ‘to attack’ [6]. Aggression is any behavior that directly harms someone; everyone can do it [7]. Aggression can happen in different ways. For example, verbal and physical aggression represent instrumental or behavioral elements, and hostility, revelation, and anger demonstrate the cognitive aspects of aggression [8]. However, aggression towards nurses arises from various sources, such as other nurses, relatives, doctors or allied health professionals, and patients [6].

Personal experience of violence in the workplace may cause serious consequences for HCWs, patients, patient care, and the organization. Exposure to traumatic situations over the nursing career and failure to control these experiences lead to poor patient care; the complications manifest as fatigue and exhaustion [9–11]. Constant exposure leads to physical weakness, feeling angry, cynical, pessimistic, or feeling trapped, which could cause other complications such as depression and anxiety [12]. The nurse may require a response to aggression via changing the job, absenteeism from work, or leaving the nursing profession altogether. The potential of aggression and violence against HCWs is not a new phenomenon that leads to occupational anxiety; it has been reported as a frequent event [9], especially in mental health care, emergency departments, and other emergency services [13]. In the mentioned cases, the most frequently reported occupational aggressions had been imposed by patients [14]. In addition, other studies have identified the risk of aggression or violence in the workplace between healthcare employees [15]. There is, however, a lack of studies on how spirituality impacts aggression perpetration [16].

Spiritual health is considered the fourth dimension of health; its effects on bio-psycho-social health are studied worldwide [17]. Moberg first introduced the concept of spiritual health as spiritual well-being, which has always been one of the important issues in nursing. Florence Nightingale believes that spiritual care is an integral part of human needs and is necessary for healing [18]. However, spiritual health is defined as the feeling of acceptance and a positive understanding of the two-way relationship with a supernatural and superior force, which creates peace and tranquility in souls. Spiritual health can affect many psychological variables, one of which is avoiding aggression and violent behaviors [19].

As per the recent models of aggression, it is said that aggressive behaviors are the consequence of (i) personal factors such as personality traits, history of abuse, etc., (ii) situational factors such as burnout, frustration, threatening stimuli, etc., and (iii) internal states such as cognition situation [20, 21]. In addition, recent evidence indicates the importance of spirituality as a coping skill and psychological well-being maintainer for both patients and HCWs during the COVID-19 pandemic [22]. Spirituality has also been found to help HCWs combat stress, encourage recovery and resilience, and reduce burnout [23]. For instance, McSherry and Jamieson reported that 83% of nurses agreed that spiritual care and spirituality as fundamental aspects of nursing, and 90% believed that providing spiritual care enhances the overall quality of nursing care [24]. As reported in another study, nurses who had a higher level of spiritual health paid more attention and love to the patients and had more spiritual care for patients [25]. Therefore, it is hypothesized that nurses with higher spirituality will have lower aggression levels.

As stated by Panzini et al. [26], spirituality is an approach that manages people’s life-threatening conditions. It is also a mechanism to fight against occupational stress and burnout syndrome in many professions, including HCWs [27]. Furthermore, spirituality provides peace of mind and enhances the sense of purpose and life meaning [28], which can help reduce aggression. However, despite the importance of mental health in nursing care, as per our literature review, no study has been conducted about the spiritual health of nurses in relation to aggression during the COVID-19 pandemic. Accordingly, to the proposed hypothesis that spirituality appears to reduce aggression, and in this study, the levels of aggression and the effects of spirituality posed to aggression among the Iranian nurses working in the COVID-19 wards were investigated. In addition, the relationships between socio-demographics, and spirituality and aggression were also investigated.

## 2 Methods

### 2.1 Sampling and participants

This study design was a cross-sectional study conducted among Iranian nurses. The study population of this study is the COVID-19 ward working nurses, who were selected by a random sampling method. The data collection time was June to August 2021. At the time of this study coincided with the peak of the COVID-19 pandemic and it was the fifth peak of the disease in Iran. The number of patients who were referred to selected hospital centers during the day was very high, and the situation was as extreme as sometimes the bed, especially the ICU bed, was challenging to allocate for patients. However, four hospitals (i.e., Khatam Hospital, Imam Hospital, Shohada Hospital and Samen Gulogah Hospital) were purposively selected from the east of the Mazandaran province to collect data. The inclusion criteria for participation in this study included: (a) registered nurses, (b) nurses involved in COVID-19 wards, and (c) voluntary participation.

$$\text{n}=\frac{2\sigma^{2}{\left({\text{z}}_{1-\frac{\alpha }{2}}+{\text{z}}_{1-\beta }\right)}^{2}}{{\text{d}}^{2}}=\frac{2{\left(1.50\right)}^{2}{\left(1.96+0.90\right)}^{2}}{0.184}=200.04$$

The sample size, according to the population size and based on the following equation [29] and considering σ = 1.5, d^2^ = 0.184, α = 1.96, and power = 0.90, was equal to 200 people selected. A total of 200 participants among 385 nurses working in the COVID-19 ward in the hospitals were chosen using a simple randomization approach to data collection. The response rate was 100%. Data were obtained by setting up an online questionnaire, and the required descriptions were given to complete the questionnaires. After signing an informed consent form, the nurses participated in this study and returned it to the researchers through e-mail. The data collection timeline was about three months, and the duration of filling out each questionnaire was about 10 minutes.

### 2.2 Ethical consideration

Before conducting this study, the protocol was approved by the Ethics Committee of Mazandaran University of Medical Sciences (Reference: MAZUMS.REC.1399.8763). Furthermore, a letter of introduction was obtained from the research deputy of Mazandaran University of Medical Sciences, which was submitted to the heads Hospital while the study was conducted. In order to ensure the confidentiality of the participants’ information, the names of the participants were not mentioned in the questionnaire, and they were assured that their information was for research purposes only. Finally, after signing an informed consent form, the nurses participated in this study and returned the questionnaire through e-mail.

### 2.3 Measures

#### 2.3.1 Socio-demographic measures

Participants’ socio-demographic information was collected based on gender (male, female), marital status (married, unmarried), education level (bachelor, associate degree, higher degree), working section (ICU or CCU, surgery unit, internal medicine, emergency, pediatrics, others), work experience (less than one year, between one to ten year, more than ten years). English version of the questionnaire used in this study was provided as the S2 File.

#### 2.3.2 Palutzian-Ellison spiritual well-being questionnaire

The Spiritual Well-being Questionnaire was used to assess spirituality, which was developed by Palutzian and Ellison [30]. This scale consists of 20 items which are divided into two dimensions entitled ‘‘Religious health” and ‘‘Existential health”. The items are responded to with a 6-point Likert scale, where a score of 6 means totally agree and a score of 1 means totally disagree. The overall score of the questionnaire is 20–120. A higher score means a better spiritual health condition [30]. The prior studies confirmed the Persian version of this scale’s validity [31]. The reliability coefficient of the questionnaire was calculated to be 0.82, by Cronbach’s alpha [32, 33]. However, the validity and reliability were reassessed; Cronbach’s alpha coefficient was 0.86 in this study.

#### 2.3.3 Buss-Perry aggression questionnaire

The Aggression Questionnaire was used to assess aggression, which was developed by Buss and Perry [34]. This questionnaire has 29 questions and measures four aspects of aggression (i.e., physical, verbal, anger, and hostility) and, in general, the level of individual aggression. This scale includes physical aggression (9 items), verbal aggression (5 items), anger (7 items), and hostility (8 items) [35]. The Cronbach’s alpha coefficient (internal consistency coefficient) for the components of the questionnaire was 0.89 [36]. The Persian version of this scale’s validity was confirmed by the prior studies [37]. All components of this scale are ranked based on a 5-option Likert (totally agree to totally disagree) [36]. The validity and reliability were also reassessed; the Cronbach’s alpha coefficient was 0.90 in this study.

### 2.4 Data analysis

Descriptive statistics, including frequency, percentage, mean and standard deviation, were reported. Inferential statistics such as t-test and one-way ANOVA were conducted depending on the samples of the socio-demographic variables. Further, the Pearson correlation coefficient was used to determine the relationship between aggression, and spiritual health. In addition, multiple linear regression was used to see the level of aggression based on spiritual health and demographic variables. Data were normally distributed, and a multi-collinearity test was also done and all independent variables had a Variance Inflation Factor (VIF) less than 3. All tests were analyzed through the SPSS statistics software V. 21 at a significance level of <0.05.

## 3 Results

### 3.1 Socio‐demographic characteristics

The mean age of 200 nurses was 31.49±6.88 years. All participants were Muslim. Among the participants, 78.5% were female, and 21.5% were male. In terms of marital status, 26.5% were single, and 73.5% were married. Most of them had a bachelor’s degree (85.0%) and 1–10 years of work experience (33.5%) and worked emergency department (31.5%) (Table 1). However, the descriptive indices of spiritual health and aggression, including mean, SD, minimum, and maximum, are shown in Table 2. Results showed that participants had a mean spirituality score of 67.21 ± 12.84 out of 120, whereas the mean score of aggression was 51.77 ± 10.96 out of 116 (Table 2). A comparison of mean scores of spiritual health and aggression based on the socio-demographic variables was shown in Table 1, and none of the variables were significantly associated.

**Table 1:** Distribution of the socio-demographic variables with spiritual health and aggression.

Variables	*n*, %	Spirituality	Aggression
**Gender**			
Male	43, 21.5%	67.44 ± 11.82	51.84 ± 10.56
Female	157, 78.5%	67.15 ± 13.14	51.75 ± 11.09
**Marital status**			
Married	147, 73.5%	67.28 ± 12.55	52.30 ± 11.26
Single	53, 26.5%	67.04 ± 13.73	50.28 ± 9.98
**Education level**			
Bachelor	170, 85.0%	6.06 ± 12.91	51.96 ± 10.99
Associate degree	19, 9.5%	60.90 ± 11.27	50.88 ± 11.31
Higher bachelor	11, 5.5%	65.08 ± 11.86	50.32 ± 10.51
**Working section**			
ICU or CCU	25, 12.5%	65.54 ± 13.75	49.99 ± 10.10
Surgery unit	45, 22.5%	69.00 ± 15.62	52.56 ± 13.67
Internal medicine	31, 15.5%	66.89 ± 11.57	52.81 ± 7.65
Emergency	63, 31.5%	66.99 ± 12.08	50.82 ± 10.40
Pediatrics	12, 6.0%	67.71 ± 9.17	54.35 ± 8.86
Others	24, 12%	66.51 ± 11.94	51.97 ± 12.49
**Work experience**			
Less than 1 year	18, 9.0%	63.56 ± 19.05	52.46 ± 10.18
1–10 years	108, 54.0%	67.69 ± 12.45	50.61 ± 10.47
More than 10 years	74, 37.0%	67.41 ± 11.56	53.29 ± 11.74

**Table 2:** Description of the spirituality and aggression.

Variables	Mean ± SD	Minimum score	Maximum score
**Age**	31.74±6.49	21	48
**Spiritual health**	67.21±12.84	24	115
Religious health	32.76±7.13	12	58
Existential health	34.28±6.09	15	60
**Aggression**	51.77±10.96	29	107
Physical Aggression	17.80±4.84	8	33
Verbal Aggression	8.71±1.69	6	21
Anger	13.42±2.13	7	26
Hostility	15.28±3.33	7	31

### 3.2 Correlation coefficient of aggression and spiritual health

According to the results of Table 3, there was a significant negative correlation between aggression and spiritual health (r = —.285, *p* < .01). That means, with increasing spiritual health scores, the level of aggression decreased. The correlations of aggression and spirituality subscales can be found in Table 3.

**Table 3:** Correlation coefficient between aggression and spirituality.

Variables	1	2	3	4	5	6	7	8
**Spiritual health (1)**	1							
Religious health (2)	.657**	1						
Existential health (3)	.678**	.326**	1					
**Aggression (4)**	-.285**	-.110	-.161*	1				
Physical aggression (5)	-.20**	-.090	-.122	.941**	1			
Verbal aggression (6)	-.266**	-.092	-.173*	.954*	.815**	1		
Anger (7)	-.263**	-.169*	-.198*	.659**	.488**	.688**	1	
Hostility (8)	-.255**	-.104	-.102	.889**	.831**	.824**	.394**	1

* *p* <0.05;

** *p*< 0.01

### 3.3 Factors associated with aggression

The results in Table 4, show factors associated with aggression based on two models. In the first model, only considered spiritual health and a total of 7.7% variance was explained. After adding socio-demographic factors with spiritual health in the final model, about 11.4% variance can be explained. Based on the final model, spiritual health [-.264], age [-.374], and working experience [4.156] were found as significant factors of aggression.

**Table 4:** Associated factors of aggression based on spirituality and socio-demographics.

Model		Unstandardized Coefficients		Standardized Coefficients Beta	t	Sig.
		B	Std. Error			
1	(Constant)	67.580	4.010		16.853	< .001
	Spiritual health	-.237	.058	-.278	-4.047	< .001
2	(Constant)	77.815	8.078		9.633	< .001
	Spiritual health	-.264	.059	-.310	-4.452	< .001
	Age	-.374	.191	-.222	-1.953	.052
	Gender	-1.123	1.893	-.042	-.593	.554
	Marital status	-2.139	1.887	-.087	-1.134	.258
	Education level	-1.401	1.497	-.067	-.936	.351
	Working section	.113	.506	.016	.223	.824
	Work experience	4.156	1.974	.236	2.105	.037

## 4 Discussion

The COVID-19-related stressors have appeared to increase cognition, emotion, and physiological state problems, increasing the threat risk, leading to fight-or-flight tendencies, and elevating anger-reactive physical aggression levels [38]. Evidence supports that aggression has been raised during the COVID-19 pandemic; for instance, al-Mamun et al. observed sexual violence increment in Bangladesh [3], similar to other countries such as Russia [39], and the United States [40]. Of the healthcare professionals, about 65.5% reported being exposed to workplace violence, mainly verbal violence in 52.0% during the COVID-19 pandemic, as per a Jordanian study [41]. In the same study, the contributing factors of violence in the workplace have been reported, such as intense workload, high patient expectations, substance abuse by the patient, long waiting period, rejection of demands that cannot be accepted, and sensational reports from media, inadequate security, etc. Another study reported that nursing technicians or assistants were at higher risk of violence during the pandemic [42]. About 44.4% of the nurses have experienced physical violence during the pandemic, while 67.8% for verbal abuse [43]. Those nurses who worked for COVID-19 patient’s care were at 2.18 times and 2.10 times higher risk of physical violence and verbal abuse, respectively [43]. All of that evidence is based on type II level of aggression, that is, aggression imposed on healthcare professionals.

Despite that evidence, there is a lack of information about the nurses’ aggression toward others and the role of spirituality in aggression, especially during the COVID-19 pandemic. Based on the prior works, it is speculated that spirituality can be beneficial in controlling behaviors like aggression [44–46]. The findings of this study also depicted that spirituality can be of the factors reducing the level of aggression among the Iranian nurses working in the COVID-19 wards. It should be noted that religious health of the spirituality subdimension was not significantly correlated with aggression, although existential health was found to be negatively significantly correlated with the aggressiveness of the nurses. However, based on this study, a transcendent human being can be seen if a person can communicate ethically with others and help them in all circumstances. Therefore, it is likely that overall spiritual health training and enhancement can help decrease violent and aggressive behaviors.

The present study found a reverse relationship between spirituality and aggression. Similarly, spirituality has a positive role in reducing the aggression of undergraduate students, as reported in prior Iranian studies [45]. Furthermore, studies from other countries, for example, Jordan, indicated a significant negative correlation between the total scores of spiritual well-being and aggression [46]. Therefore, spiritual health encourages nurses to be more comfortable and relaxed, which allows them to provide more forgiveness and compassion, which may release negative emotions such as anger and hostility and decrease the levels of aggression. That is because of the fact, spirituality is recognized as a coping strategy and resource that assists people in finding meaning in their lives and safely getting through the difficult situations they face [47].

The present study had a few limitations to be noted. First, the association between nurses’ spiritual health and aggression was only investigated. Other professional nursing factors, namely attitude toward spiritual care, professional commitment, and caring, may also be correlated with spiritual health; future studies should address them. Second, the participants from four hospitals of one providence were selected; therefore, the generalization of the study results may be limited inside and outside of the country. In addition, other issues like memory recall bias, self-reported data and its inherent bias can be placed while conducting this study, limiting the findings. Third, the measure of aggression used the Buss & Perry Aggression Questionnaire version, where there are some concerns because factor analysis has not verified the original factors. The revised version has rectified this issue with a five-factor solution, and it is suggested to consider the preferable version to use for further studies [48]. Finally, the findings of this study are based on cross-sectional observation; therefore, causal relationships should be determined based on robust methodologies accounting for other shortcomings of this study.

## 5 Conclusions

In general, nurses who consider spirituality their standard in life perform their actions based on knowledge and awareness; they can also reduce negative emotions, such as aggression. In addition, spiritually healthy nurses may be more powerful in helping patients by providing caring or spiritual support. Therefore, it is recommended that future researches focus on developing an intervention program to improve nurses’ attitudes toward spiritual health, the feasibility and potential efficacy of promoting such a spiritual health program, and the relationship between nurses’ spiritual health and quality of care. Healthcare managers, planners and nursing educators need to reform policies with a focus on spiritual care education, providing the ground for promoting spirituality and a positive attitude towards it.

## Supporting information

S1 File Data. (DOCX)

S2 File Questionnairre. (DOCX)

S3 File Inclusivity in global research. (SAV)
