# Supplementary material for: The relationship between spirituality and aggression in the workplace during the COVID-19 pandemic: A cross-sectional study among Iranian nurses
Source: PLoS One. 2022 Dec 21;17(12):e0279247. doi: 10.1371/journal.pone.0279247 (PMC9770337; doi:10.1371/journal.pone.0279247)
Supplement: S2 File — (DOCX) [file pone.0279247.s002.docx]

1. Age: ………………….
2. Gender: Male۝ Female ۝
3. Working section: ………………
4. Marital status: Married ۝ Single ۝
5. Education level: Bachelor ۝ Associate degree ۝ Higher bachelor ۝
6. Work experience: ……………………….

|  | **Buss-Perry Aggression Questionnaire** | Totally disagree | disagree | No idea | agree | Totally agree |
| --- | --- | --- | --- | --- | --- | --- |
| 1 | Once in a while I can’t control the urge to strike another person. |  |  |  |  |  |
| 2 | Given enough provocation, I may hit another person. |  |  |  |  |  |
| 3 | If somebody hits me, I hit back. |  |  |  |  |  |
| 4 | I get into fights a little more than the average person. |  |  |  |  |  |
| 5 | If I have to resort to violence to protect my rights, I will. |  |  |  |  |  |
| 6 | There are people who pushed me so far that we came to blows. |  |  |  |  |  |
| 7 | I can think of no good reason for ever hitting a person (R). |  |  |  |  |  |
| 8 | I have threatened people I know. |  |  |  |  |  |
| 9 | I have become so mad that I have broken things. |  |  |  |  |  |
| 10 | I tell my friends openly when I disagree with them. |  |  |  |  |  |
| 11 | I often find myself disagreeing with people. |  |  |  |  |  |
| 12 | When people annoy me, I may tell them what I think of them. |  |  |  |  |  |
| 13 | I can’t help getting into arguments when people disagree with me. |  |  |  |  |  |
| 14 | My friends say that I’m somewhat argumentative. |  |  |  |  |  |
| 15 | I flare up quickly but get over it quickly. |  |  |  |  |  |
| 16 | When frustrated, I let my irritation show. |  |  |  |  |  |
| 17 | I sometimes feel like a powder keg ready to explode. |  |  |  |  |  |
| 18 | I am an even-tempered person (R). |  |  |  |  |  |
| 19 | Some of my friends think I’m a hothead. |  |  |  |  |  |
| 20 | Sometimes I fly off the handle for no good reason. |  |  |  |  |  |
| 21 | I have trouble controlling my temper. |  |  |  |  |  |
| 22 | I am sometimes eaten up with jealousy. |  |  |  |  |  |
| 23 | At times I feel I have gotten a raw deal out of life. |  |  |  |  |  |
| 24 | Other people always seem to get the breaks. |  |  |  |  |  |
| 25 | I wonder why sometimes I feel so bitter about things. |  |  |  |  |  |
| 26 | I know that "friends" talk about me behind my back. |  |  |  |  |  |
| 27 | I am suspicious of overly friendly strangers. |  |  |  |  |  |
| 28 | I sometimes feel that people are laughing at me behind me back. |  |  |  |  |  |
| 29 | When people are especially nice, I wonder what they want. |  |  |  |  |  |

|  | **Palutzian-Ellison Spiritual Well-being Questionnaire** | Totally disagree | disagree | Partly disagree | Partly agree | agree | Totally agree |
| --- | --- | --- | --- | --- | --- | --- | --- |
| 1 | I don’t find much satisfaction in private prayer with God. |  |  |  |  |  |  |
| 2 | I don’t know who I am, where I came from, or where I’m going |  |  |  |  |  |  |
| 3 | I believe that God loves me and cares about me. |  |  |  |  |  |  |
| 4 | I feel that life is a positive experience |  |  |  |  |  |  |
| 5 | I believe that God is impersonal and not interested in my daily  situations |  |  |  |  |  |  |
| 6 | I feel unsettled about my future |  |  |  |  |  |  |
| 7 | I have a personally meaningful relationship with God. |  |  |  |  |  |  |
| 8 | I feel very fulfilled and satisfied with life |  |  |  |  |  |  |
| 9 | I don’t get much personal strength and support from my God. |  |  |  |  |  |  |
| 10 | I feel a sense of well-being about the direction my life is headed in |  |  |  |  |  |  |
| 11 | I believe that God is concerned about my problems |  |  |  |  |  |  |
| 12 | I don’t enjoy much about life |  |  |  |  |  |  |
| 13 | I don’t have a personally satisfying relationship with God |  |  |  |  |  |  |
| 14 | I feel good about my future |  |  |  |  |  |  |
| 15 | My relationship with God helps me not to feel lonely |  |  |  |  |  |  |
| 16 | I feel that life is full of conflict and unhappiness. |  |  |  |  |  |  |
| 17 | I feel most fulfilled when I’m in close communion with God. |  |  |  |  |  |  |
| 18 | Life doesn’t have much meaning. |  |  |  |  |  |  |
| 19 | My relation with God contributes to my sense of well-being |  |  |  |  |  |  |
| 20 | I believe there is some real purpose for my life |  |  |  |  |  |  |
